# Preservation versus nonpreservation of the left colic artery in anterior resection for rectal cancer: a propensity score-matched analysis

**DOI:** 10.1186/s12893-022-01614-y

**Published:** 2022-05-10

**Authors:** Huichao Zheng, Fan Li, Xingjie Xie, Song Zhao, Bin Huang, Weidong Tong

**Affiliations:** Department of General Surgery, Daping Hospital, Army Medical University, No. 10, Changjiang Branch Road, Daping, Yuzhong District, Chongqing, 400042 China

**Keywords:** Rectal cancer, Left colic artery, Anterior resection, Anastomotic leakage, Inferior mesenteric artery, Surgery

## Abstract

**Background:**

Preserving the left colic artery (LCA) during anterior resection for rectal cancer is controversial, and robust evidence of the outcomes of LCA preservation plus apical lymph node dissection is lacking. The purpose of this study was to investigate the impact of LCA preservation plus apical lymph node dissection surgery on anastomotic leakage and number of harvested lymph nodes.

**Methods:**

Patients who underwent laparoscopic or robotic anterior resection for rectal cancer between September 2017 and May 2020 were retrospectively assessed. The patients were categorized into two groups: preservation of LCA and nonpreservation of LCA. A one-to-one propensity score-matched analysis was performed to decrease confounding. The primary outcome was anastomotic leakage within 30 days after surgery. The secondary outcomes were number of harvested lymph nodes, 3-year overall survival, and 3-year disease-free survival.

**Results:**

A total of 216 patients were eligible for this study, and propensity score matching yielded 60 patients in each group. Anastomotic leakage in the LCA preservation group was significantly lower than that in the LCA nonpreservation group (3.3% vs. 13.3%, P = 0.048). No significant differences were observed in blood loss, operation time, intraoperative complications, splenic flexure mobilization, total number of harvested lymph nodes, number of positive lymph nodes, time to first flatus, or postoperative hospital stay. Kaplan–Meier survival analysis showed a 3-year disease-free survival of 85.7% vs. 80.5% (P = 0.738) and overall survival of 92.4% vs. 93.7% (P = 0.323) for the preservation and nonpreservation groups, respectively.

**Conclusion:**

LCA preservation plus apical lymph node dissection surgery for rectal cancer may help reduce the incidence of anastomotic leakage without impairing the number of harvested lymph nodes. Preliminary results suggest that 3-year disease-free survival and overall survival rates may not differ between the two types of surgery, but studies with larger sample sizes are needed to confirm these conclusions.

*Trial registration* ClinicalTrials.gov, NCT03776370. Registered 14 December 2018—Retrospectively registered, https://clinicaltrials.gov.

## Introduction

Anastomotic leakage (AL) is one of the most serious postoperative complications after anterior resection for rectal cancer. It is reported to be associated with a higher mortality rate, longer hospital stay, higher local recurrence rate, and poorer survival outcomes [1–3]. The two key factors in preventing AL are ensuring that the anastomosis is tension-free and has a sufficient blood supply [4–6]. It has been suggested that anastomotic blood supply is correlated with the ligation level of the inferior mesenteric artery (IMA), although this possibility is controversial [7–9].

Generally, there are two strategies for ligation of the IMA: (1) high ligation, in which the IMA is ligated approximately 1 cm from its origin, with nonpreservation of the left colic artery (LCA), and (2) low ligation, in which the IMA is ligated below the origin of the LCA, with preservation of the LCA. Preservation of the LCA during rectal cancer surgery is controversial [10, 11], and current clinical practice guidelines do not specifically address this issue. On the one hand, the impact of preserving the LCA on the development of AL in rectal cancer patients following anterior resection remains controversial [12–14]. Another point of debate is whether LCA preservation affects lymph node (LN) dissection and oncological outcomes [15, 16]. Since the benefits of LCA preservation are debatable, robust evidence of the outcomes of LCA preservation plus apical LN dissection is lacking. This propensity score matching study was designed to investigate the impact of LCA preservation plus apical LN dissection surgery on AL and LN harvesting.

## Materials and methods

### Patient selection

**Fig. 1:**
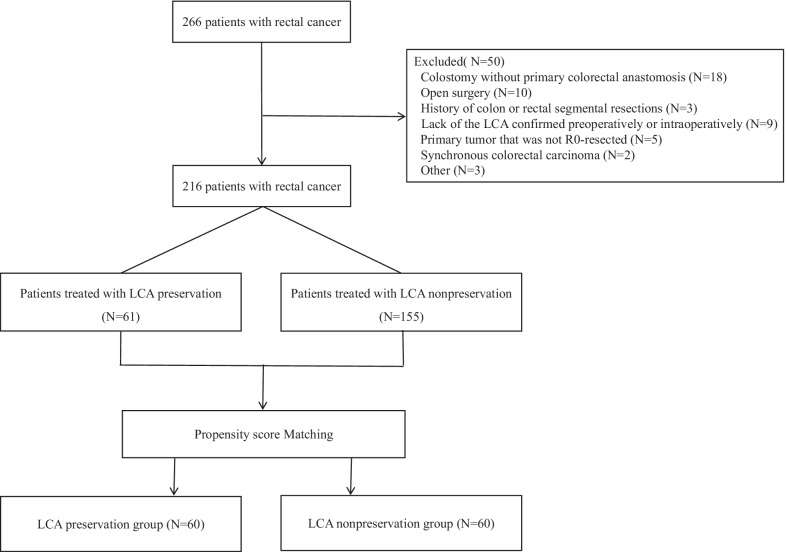
CONSORT flow chart of the study. *LCA* left colic artery

Patients who underwent laparoscopic or robotic anterior rectal resections carried out by two surgeons between September 2017 and May 2020 at Daping Hospital were retrospectively assessed. The two surgeons perform at least 100 rectal cancer operations every year and have completed their learning curve in laparoscopic and robotic colorectal operations. The inclusion criteria were as follows: (1) 18 years of age and over; (2) laparoscopic or Da Vinci Si robot-assisted anterior resection for rectal cancer; and (3) American Society of Anesthesiologists (ASA) grade less than or equal to III. The exclusion criteria were as follows: (1) synchronous colorectal carcinoma; (2) emergency surgery; (3) primary tumor that was not R0-resected; (4) history of colon or rectal segmental resections; and (5) lack of the LCA confirmed preoperatively or intraoperatively. Figure 1 shows the study flow chart. This study was approved by the ethics committee of Army Medical University Daping Hospital (Project ID: 201855). The study was performed in accordance with the ethical standards laid down in the 1964 Declaration of Helsinki and its later amendments or comparable ethical standards. The trial protocol is available from the corresponding author.

### Data collection

The clinicopathological data of the patients were collected prospectively and reviewed retrospectively. The baseline data included age, sex, BMI, and ASA classification. The tumor characteristics included TNM stage, tumor differentiation, total number of harvested LNs, and number of positive LNs. Perioperative outcomes included operation time, estimated blood loss, conversion rate, splenic flexure mobilization, duration of peritoneal drainage tube, intraoperative complications, postoperative complications, time to first flatus, and postoperative hospital stay. Pathologic staging of the tumors was based on the staging criteria of the American Joint Committee on Cancer 7th edition.

### Surgical technique

**Fig. 2:**
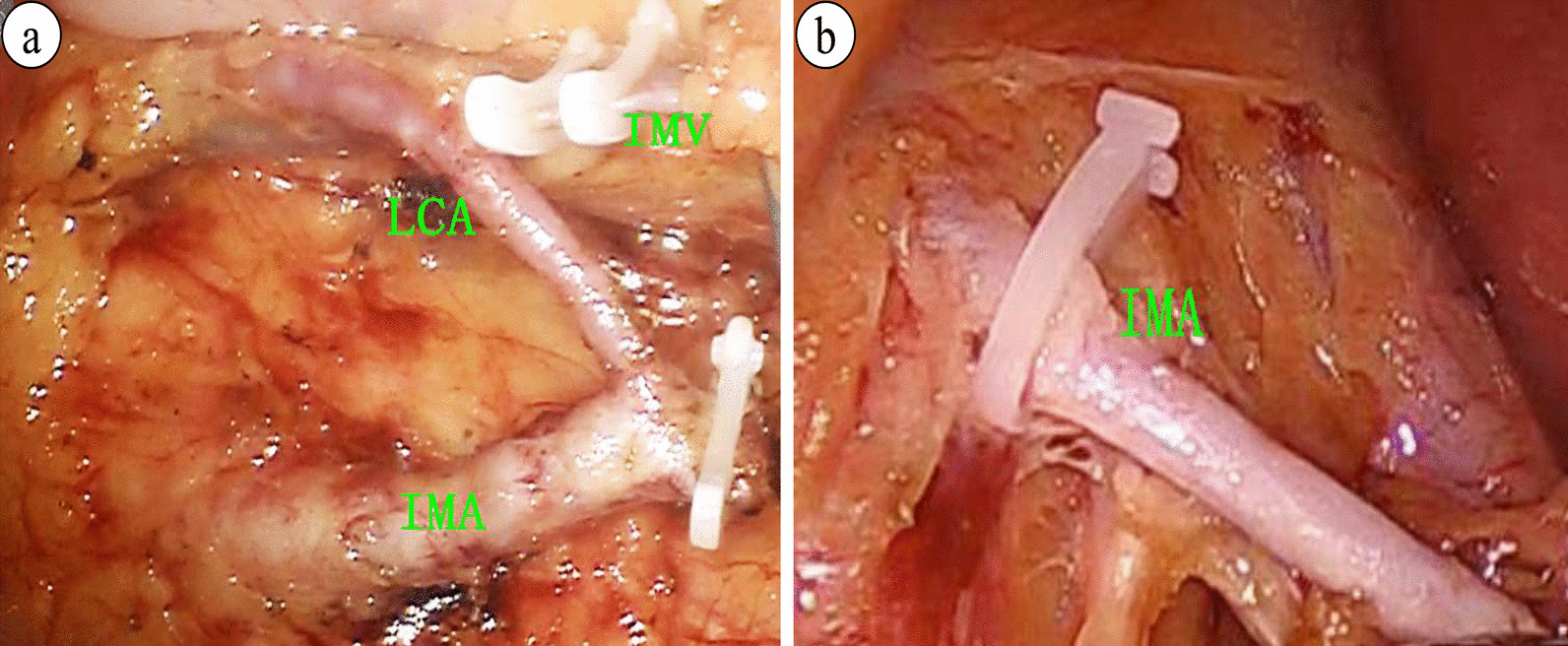
Preservation or nonpreservation of the left colic artery in anterior resection for rectal cancer. **a** Preservation of the left colic artery: the IMA was ligated below the origin of the LCA; **b** Nonpreservation of the left colic artery: the IMA was ligated 1 cm from its origin. *IMA* inferior mesenteric artery; *LCA* left colic artery; *IMV* inferior mesenteric vein

All the patients were placed in the lithotomy position and were under general anesthesia. For details regarding the surgical procedures, refer to the Chinese guidelines on laparoscopic or robotic-assisted radical resection surgery for colorectal cancer. The decision to preserve the LCA was made by the operating surgeon. For the LCA preservation group (Fig. 2a), the IMA, LCA, sigmoid artery (SA) and superior rectal artery (SRA) were identified, and then the LCA was preserved simultaneously with ligations of the SA, SRA and inferior mesenteric vein (IMV). Furthermore, lymphadenectomy around the root of the IMA was performed. For the LCA nonpreservation group (Fig. 2b), the IMA was ligated 1 cm from its origin with lymphadenectomy around the IMA, and the IMV was ligated at the same level. All the patients underwent total mesorectal excision or tumor-specific mesorectal excision. The surgical specimen was removed through a 5 cm abdominal incision. Most anastomoses were performed by using the double stapling technique, except for ultralow anterior resection with a hand-sewn coloanal anastomosis. If the colon was not free, the splenic flexure of the colon was mobilized. After the completion of anastomosis, the pelvic cavity was washed out, and an air leakage test was conducted. According to the patient's medical history and the quality of the anastomosis, the surgeons decided whether to perform protective ileostomy. Finally, the abdominal drainage tube was placed in the pelvic cavity and fixed through the abdominal wall.

### Neoadjuvant therapy, adjuvant therapy and patient follow-up

Preoperative magnetic resonance imaging of the pelvis was performed to identify the clinical stage. According to the National Comprehensive Cancer Network guidelines (version 3, 2016), preoperative neoadjuvant therapy is recommended for tumors located within 12 cm from the anal verge and tumors of clinical stage T3 or T4 or those with LN positivity. The interval between the completion of neoadjuvant therapy and surgery was 6–8 weeks. Postoperative adjuvant therapy is recommended for stage II patients with risk factors and stage III or stage IV patients after surgery. These patients received a 5-fluorouracil-based chemotherapy regimen.

Recurrence, metastasis and survival information was obtained by outpatient visits or telephone follow-up. Specifically, the patients were followed up at 3-month intervals for the first 2 years and 6-month intervals thereafter; serum carcinoembryonic antigen levels, colonoscopy results, and computer tomography results of the chest and abdomen were assessed.

### Outcome assessment

Our primary outcome was AL within 30 days after surgery and was assessed according to the definition and severity grading proposed by the International Study Group of Rectal Cancer in 2010 [17]: grade A requires no active therapeutic intervention; grade B requires nonsurgical therapeutic intervention such as antibiotics or percutaneous drainage; and grade C requires operative reintervention. In the present study, AL was referred to as symptomatic AL (grades B and C); asymptomatic AL (grade A) was not considered because no active therapeutic intervention was required. AL was determined by digital rectal examination, abdominal computer tomography scan, clinical symptoms, or surgery when necessary. Postoperative complications were graded based on the Clavien–Dindo classification [18, 19], and we grouped complications into major (Clavien–Dindo III–V) and minor (Clavien–Dindo I–II) complications. The secondary outcomes were the number of harvested LNs, 3-year overall survival (OS), and 3-year disease-free survival (DFS).

### Statistical analysis

A one-to-one propensity score-matched analysis was performed to decrease confounding by using IBM SPSS 25.0, and the caliper was calculated to be 0.03. The patients in this cohort study were grouped based on the following variables: age, sex, body mass index (BMI), ASA classification, tumor location, pathological TNM stage, surgical approach (laparoscopic or robotic), transanal total mesorectal excision (TaTME), protective ileostomy, and preoperative neoadjuvant therapy. A total of 216 patients were eligible for this study, and propensity score matching yielded 60 patients in each group.

Ten patients had missing data, and the proportion of missing data for each variable was less than 5%. Multiple imputation using chained equations was performed to account for the missing data [20]. To control confounding, subgroup analysis was performed according to the presence or absence of a protective ileostomy. Continuous variables with a normal distribution are presented as the mean ± standard deviation, and intergroup comparisons were analyzed by Student’s *t* test. Continuous variables with a skewed distribution are described as medians and ranges, and comparisons between two groups were performed by using the Mann–Whitney U test. Categorical variables are presented as absolute numbers and percentages. The chi-square test and Fischer’s exact test were used to compare categorical variables, and the Mann–Whitney U test was used to evaluate the significance of differences between rank variables. OS and DFS were analyzed by using the Kaplan–Meier method, and comparison of survival between the groups was performed by the log rank test. Five patients were lost to survival follow-up and were excluded from the survival analysis. P values were derived from two-tailed tests, and P < 0.05 was considered statistically significant. Statistical analysis was performed using IBM SPSS Statistics, version 25.

## Results

### Patient characteristics

Data from a total of 266 patients were collected, and 50 patients were excluded. Before case matching, the proportion of patients who underwent the TaTME procedure in the LCA preservation group was lower than that in the LCA nonpreservation group (18.0% vs. 34.2%, P < 0.05), and more patients in the LCA preservation group underwent robotic surgery. After 1:1 propensity score matching, the patients were matched into an LCA preservation group of 60 patients and an LCA nonpreservation group of 60 patients. Both groups were well balanced in terms of age, sex, BMI, ASA classification, tumor location, TNM stage, surgical approach (laparoscopic or robotic), TaTME, protective ileostomy, and preoperative neoadjuvant therapy. There were no significant differences in the baseline characteristics of the groups after matching. Patient clinical characteristics before and after propensity score matching are shown in Table 1.

**Table 1:** Patients’ clinical characteristics before and after PSM

Parameter	Before PSM			After PSM		
	LCA preservation (n = 61)	LCA nonpreservation (n = 155)	P value	LCA preservation (n = 60)	LCA nonpreservation (n = 60)	P value
Age	59.5 ± 11.3	59.0 ± 11.0	0.784	59.5 ± 11.4	60.3 ± 10.5	0.696
Sex			0.334			0.855
Male	31 (50.8)	90 (58.1)		31 (51.7)	30 (50.0)	
Female	30 (49.2)	65 (41.9)		29 (48.3)	30 (50.0)	
BMI (kg/m^2^)	23.1 ± 3.3	23.1 ± 2.8	0.841	23.1 ± 3.3	23.2 ± 2.8	0.846
ASA classification			0.190			
I	26 (42.6)	81 (52.2)		26 (43.3)	26 (43.3)	0.843
II	28 (45.9)	61 (39.4)		27 (45.0)	29 (48.3)	
III	7 (11.5)	13 (8.4)		7 (11.7)	5 (8.4)	
Tumor location			0.719			0.752
Lower (≤ 5 cm)	16 (26.2)	54 (34.8)		16 (26.7)	14 (23.3)	
Middle (5.1–10 cm)	37 (60.7)	71 (45.8)		36 (60.0)	38 (63.4)	
Upper (10.1–15 cm)	8 (13.1)	30 (19.4)		8 (13.3)	8 (13.3)	
Pathological stage			0.643			0.866
Stage 0	4 (6.5)	9 (5.8)		4 (6.7)	5 (8.3)	
Stage 1	18 (29.5)	39 (25.2)		18 (30.0)	14 (23.3)	
Stage 2	17 (27.9)	45 (29.0)		17 (28.3)	20 (33.3)	
Stage 3	15 (24.6)	50 (32.3)		15 (25.0)	16 (26.7)	
Stage 4	7 (11.5)	12 (7.7)		6 (10.0)	5 (8.4)	
Neoadjuvant therapy	12 (19.7)	34 (21.9)	0.715	12 (20.0)	15 (25.0)	0.512
TaTME			0.019^a^			0.817
Yes	11 (18.0)	53 (34.2)		11 (18.3)	12 (20.0)	
No	50 (82.0)	102 (65.8)		49 (81.7)	48 (80.0)	
Ileostomy			0.252			1.000
Yes	33 (54.1)	97 (62.6)		33 (55.0)	33 (55.0)	
No	28 (45.9)	58 (37.4)		27 (45.0)	27 (45.0)	
Surgical approach			0.058			0.702
Laparoscope	40 (65.6)	121 (78.1)		40 (66.7)	38 (63.3)	
Robotic	21 (34.4)	34 (21.9)		20 (33.3)	22 (36.7)	

*PSM* propensity score matching; *LCA* left colic artery; *BMI* body mass index (kg/m^2^); *ASA* American Society of Anesthesiologists; *TaTME* transanal total mesorectal excision

### Surgical parameters and postoperative data

Table 2 summarizes the surgical parameters and postoperative data. No significant differences were observed in blood loss, operation time, or type of anastomosis. No intestinal perforation, bladder injury, urethral injury or other intraoperative complications were found in any of the 120 patients. Five patients underwent splenic flexure mobilization (LCA preservation vs. LCA nonpreservation: 2 vs. 3 cases, P = 0.289). There were no deaths reported within 30 days after surgery in either group. AL occurred in 10 of the 120 (8.3%) patients who underwent laparoscopic or robotic anterior resection for rectal cancer. AL in the LCA preservation group was significantly lower than that in the LCA nonpreservation group (3.3% vs. 13.3%, P = 0.048). There was one case of grade B leakage and one case of grade C leakage in the LCA preservation group, while 3 cases of grade B leakage and 5 cases of grade C leakage were diagnosed in the LCA nonpreservation group. All the patients with grade C leakage received reoperation (peritoneal irrigation and ileostomy), while the patients with grade B leakage received nonsurgical intervention (including drainage, nutrition support and anti-infection). For the patients without protective ileostomy, the AL rate of the LCA preservation group (1 of 27 [3.7%]) was lower than that of the LCA nonpreservation group (5 of 27 [18.5%]), but the difference was not statistically significant (P = 0.192). For the patients with protective ileostomy, no significant difference in the AL rate was observed between the LCA preservation group (1 of 33 [3.0%]) and the LCA nonpreservation group (3 of 33 [9.1%]; P = 0.613). AL in each subgroup based on the presence or absence of a protective ileostomy is shown in Table 3. One major complication occurred in the preservation LCA group, and 5 occurred in the nonpreservation LCA group (P = 0.209). Five minor complications occurred in the preservation LCA group, and 8 occurred in the nonpreservation LCA group (P = 0.378). No significant differences were observed in anastomotic bleeding, wound infection, time to first flatus, postoperative hospital stay, or postoperative adjuvant therapy between the two groups. The reoperation rate of the LCA preservation group (1.7%) was lower than that of the LCA nonpreservation group (8.3%), but the difference was not statistically significant (P = 0.209).

**Table 2:** Surgical parameters and postoperative data

Characteristics	LCA preservation (n = 60)	LCA nonpreservation (n = 60)	P value
Operation time, min	192.0 ± 60.9	187.4 ± 55.7	0.668
Estimated blood loss, ml	75.3 ± 68.8	69.4 ± 75.1	0.654
Type of anastomosis			1.000
Hand-sewn anastomosis	4 (6.7)	4 (6.7)	
Double-stapling anastomosis	56 (93.3)	56 (93.3)	
Major complication, n (%)	1 (1.7)	1 (1.7)	0.209
Minor complication, n (%)	5 (8.3)	8 (15.0)	0.378
Anastomotic leakage, n (%)	2 (3.3)	8 (13.3)	0.048^a^
Ileus, n (%)	1 (1.6)	2 (3.3)	1.000
Anastomotic bleeding, n (%)	0 (0)	1 (1.7)	1.000
Time to first flatus, day	2.0 ± 0.9	1.8 ± 0.9	0.148
Reoperation, n (%)	1 (1.7)	5 (8.3)	0.209
Postoperative hospital stay, day	7 (5–28)	8 (4–48)	0.121
Duration of PDT, day	7.0 ± 2.7	7.8 ± 3.8	0.216
Postoperative adjuvant therapy, n (%)	25 (41.7)	31 (51.7)	0.272

*LCA* left colic artery; *LNs* lymph nodes; *CD* Clavien–Dindo classification; *PDT* peritoneal drainage tube;

^a^P < 0.05

**Table 3:** Anastomotic leakage in each subgroup based on the presence or absence of a protective ileostomy

Anastomotic leakage	Ileostomy			Nonileostomy		
	Group, No. (%)			Group, No. (%)		
	LCA preservation (n = 33)	LCA nonpreservation (n = 33)	P value	LCA preservation (n = 27)	LCA nonpreservation (n = 27)	P value
Present	1 (3.0)	3 (9.0)	0.613	1 (3.7)	5 (18.5)	0.192
Grade B	1 (3.0)	1 (3.0)		0 2 (7.4)		
Grade C	0	2 (6.0)		1 (3.7)	3 (11.1)	

*LCA* left colic artery

### Pathology results

Postoperative pathology results are listed in Table 4. No significant differences were observed in tumor differentiation, pathological T stage, pathological N stage, positive surgical margin, or lymphovascular and perineural invasion. The total number of harvested LNs was 13.1 ± 5.8 and 13.8 ± 4.4 in the LCA preservation and LCA nonpreservation groups, respectively (P = 0.456). The number of positive LNs was 0.9 ± 1.9 and 1.2 ± 2.1 in the LCA preservation and LCA nonpreservation groups, respectively (P = 0.345).

**Table 4:** Pathology results

Characteristics	LCA preservation (n = 60)	LCA nonpreservation (n = 60)	P value
Tumor differentiation, n (%)			0.765
Low	3 (5.0)	2 (3.3)	
Middle	54 (90.0)	55 (91.7)	
High	3 (5.0)	3 (5.0)	
Pathological T stage, n (%)			0.447
0	5 (8.3)	5 (8.3)	
1	4 (6.7)	5 (8.3)	
2	18 (30.0)	11 (18.3)	
3	21 (35.0)	25 (41.7)	
4	12 (20.0)	14 (23.4)	
Pathological N stage, n (%)			0.589
0	41 (68.3)	40 (66.6)	
1	15 (25.0)	10 (16.7)	
2	4 (6.7)	10 (16.7)	
Number of harvested LNs	13.1 ± 5.8	13.8 ± 4.4	0.456
Number of positive LNs	0.9 ± 1.9	1.2 ± 2.1	0.345
DRM positive, n (%)	0 (0.0)	0 (0.0)	1.000
CRM positive, n (%)	3 (5.0)	4 (6.7)	1.000
Lymphovascular invasion, n (%)			1.000
Absent	48 (80.0)	48 (80.0)	
Present	12 (20.0)	12 (20.0)	
Perineural invasion, n (%)			0.838
Absent	44 (73.3)	43 (71.7)	
Present	16 (26.7)	17 (28.3)	

*LCA* left colic artery; *LNs* lymph nodes; *DRM* distal resection margin; *CRM* circumferential resection margin

### Oncological outcomes

**Fig. 3:**
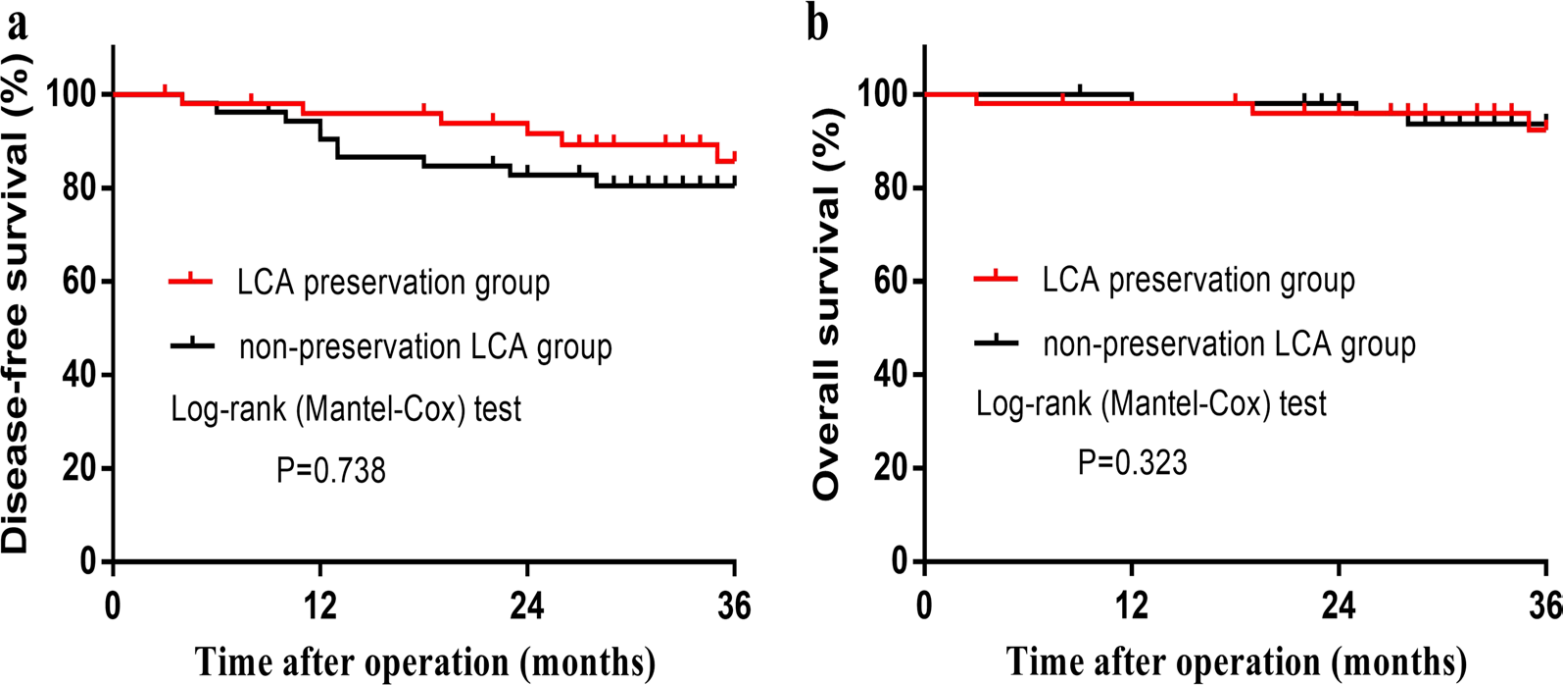
Oncological outcomes. **a** There was no significant difference in disease-free survival between the two groups (P = 0.738; log-rank test). **b** There was no significant difference in overall survival between the two groups (P = 0.323; log-rank test). *LCA* left colic artery

In total, 5 patients were lost to follow-up (3 in the LCA preservation group; 2 in the LCA nonpreservation group). The median follow-up periods were 35 (3–46) months in the LCA preservation group and 36 (9–50) months in the LCA nonpreservation group. After excluding stage IV, Kaplan–Meier survival analysis showed a 3-year estimated DFS of 85.7% vs. 80.5% (P = 0.738) and OS of 92.4% vs. 93.7% (P = 0.323) for the preservation and nonpreservation groups, respectively (Fig. 3a, b).

## Discussion

This study shows that LCA preservation plus apical LN dissection surgery has a lower AL rate after anterior resection for rectal cancer (3.3% vs. 13.3%, P = 0.048), similar to findings of previous studies [21, 22]. No significant differences were observed in blood loss, operation time, intraoperative complications, splenic flexure mobilization, time to first flatus, total number of harvested LNs, number of positive LNs, or postoperative hospital stay. These preliminary results suggest that the 3-year DFS and OS rates may not differ between the two types of surgery, but further research with larger populations is needed.

To date, the discussion of LCA preservation in rectal cancer surgery continues to be debated. The main controversy is the impact of LCA preservation on AL, apical LN dissection, and oncological outcomes. Advocates of LCA preservation contend that this technique may provide better perfusion for the proximal loop of the anastomosis to prevent AL, although this view is controversial [7, 8, 10]. Furthermore, no consensus has been reached on whether LCA preservation can prevent AL after rectal cancer surgery [12, 14, 23]. A systematic review with meta-analyses demonstrated that LCA preservation resulted in a significantly decreased incidence of AL [12]. In contrast, another recent meta-analysis showed that LCA preservation does not significantly influence the incidence of AL [14]. Different study types were included in the two meta-analyses, and methodological differences may explain the differences in the study conclusions. Our study indicated that LCA preservation was associated with a lower AL rate after anterior resection for rectal cancer (3.3% vs. 13.3%, P = 0.048). To reduce potential confounding, a stratified analysis was performed. In patients without protective ileostomy, the AL rate of the LCA preservation group was lower than that of the LCA nonpreservation group (3.7% vs. 18.5%), although the difference was not statistically significant. This finding may suggest the potential efficacy of LCA preservation in decreasing AL if a protective ileostomy has not been implemented, but larger sample size studies are needed to confirm this conclusion.

A sufficient number of harvested LNs is crucial for accurate tumor staging, and the status of LNs is a major prognostic factor in rectal cancer. Previous studies have shown that there was a trend toward worse oncological outcomes in patients with apical LN metastases [24]. Proponents of LCA nonpreservation believe that this method could help clear apical LNs and ensure the accuracy of tumor staging [25]. However, there is no strong evidence that high ligation of the IMA with LCA nonpreservation has apparent benefit for patients [15, 26, 27]. Theoretically, harvested LNs and oncological outcomes are not expected to be impaired after preservation of the LCA, as long as the procedure is complemented by dissection of the apical LNs around the IMA root. In our study, apical LN dissection was performed in the LCA preservation group. Our results showed that the total number of harvested LNs and the number of positive LNs were not different between the two groups, which is consistent with previous studies [28, 29]. Furthermore, a recent large-cohort study suggests that low ligation of the IMA did not seem to reduce the mean number of harvested positive LNs and did not affect oncologic outcomes [15]. Our study suggested that the 3-year DFS and OS rates may not differ between the two groups, but larger sample size studies are needed to confirm this conclusion.

This propensity score-matched study has several limitations. First, the sample size was small, which affected the reliability of the results at least to some extent. Second, this study was a nonrandomized controlled trial based on propensity score-matched data. Third, for the outcome of AL, the absolute numbers were low, which may limit the accuracy of determining a difference in this outcome. Fourth, defecation functions, postoperative exhaust recovery, sexual function, bladder function, and postoperative quality of life were not evaluated in this study. Fifth, this study did not construct different propensity matching models based on secondary outcomes. To overcome these limitations, further multicenter, large-sample, randomized controlled trials are needed.

## Conclusion

The results of this propensity score-matched study suggest that LCA preservation plus apical LN dissection surgery for rectal cancer may help reduce the incidence of AL without impairing the number of harvested LNs. In addition, preliminary results showed that the 3-year DFS and OS rates may not differ between the two types of surgery, but studies with larger sample sizes are needed to confirm these conclusions.

## Data Availability

The datasets generated and/or analyzed during the current study are not publicly available but are available from the corresponding author on reasonable request.

## Abbreviations

AL: Anastomotic leakage
ASA: American Society of Anesthesiologists
BMI: Body mass index
DFS: Disease-free survival
IMA: Inferior mesenteric artery
IMV: Inferior mesenteric vein
SA: Sigmoid artery
SRA: Superior rectal artery
LNs: Lymph nodes
LCA: Left colic artery
OS: Overall survival
TaTME: Transanal total mesorectal excision
